# MRI Waiting Time Disparities in Saudi Public Hospitals: A Six-Year Retrospective Study

**DOI:** 10.7759/cureus.108800

**Published:** 2026-05-13

**Authors:** Saleh Alzughaibi, Suha Kaaki, Adeeb Almohimeed, Khalid Tearo

**Affiliations:** 1 Department of Health Informatics, College of Health Sciences, Saudi Electronic University, Riyadh, SAU; 2 Division of Thoracic Surgery, Department of Surgery, College of Medicine, King Saud University, Riyadh, SAU; 3 Department of Radiodiagnostics and Medical Imaging, Prince Sultan Military Medical City, Riyadh, SAU; 4 Department of Radiological Sciences, College of Applied Medical Sciences, King Saud University, Riyadh, SAU

**Keywords:** emergency imaging, healthcare equity, mri waiting time, non-emergency imaging, scheduling disparities

## Abstract

Background: Timely access to magnetic resonance imaging (MRI) is essential for accurate diagnosis and effective patient management. Prolonged waiting times can compromise care quality and may signal inefficiencies or inequities within imaging services. Evidence on MRI waiting time patterns in Saudi Arabia's public healthcare system remains limited. This study aimed to quantify MRI waiting times and compare differences across urgency categories, patient demographics, and referring specialties in two Saudi Ministry of Health hospitals.

Methods: A retrospective cohort study was conducted using Radiology Information System data from two Ministry of Health hospitals between 2017 and 2022. Waiting time was defined as the interval between the physician's imaging request and the MRI examination date, following the Western Canada Waiting List Project definition. All completed examinations with valid timestamps were included. Descriptive statistics summarized waiting time distributions, and Mann-Whitney U tests assessed differences by urgency (emergency vs. non-emergency), gender, nationality, and referring specialty. Temporal trends were examined descriptively across annual cohorts.

Results: A total of 35,934 MRI examinations were analyzed. The overall mean waiting time was 24.8 days (SD = 32.61), with a median of 12.9 days. Emergency examinations (n = 1,128) had considerably shorter waits than non-emergency examinations (n = 34,806), with medians of 1.12 versus 13.73 days (p < 0.001). Within the non-emergency pathway, female patients waited longer than males (median = 14.94 vs. 10.76 days; p < 0.001), and Saudi nationals waited longer than non-Saudi patients (median = 14.12 vs. 4.16 days; p < 0.001); these differences may reflect case-mix, demand concentration, or scheduling dynamics rather than confirmed inequitable practices. No significant gender difference was observed in emergency cases (p = 0.410). The longest specialty-specific delays were recorded for pediatric (41.17 days), neurology (35.15 days), and urology (35.05 days) referrals. Year-to-year fluctuations were noted, with the shortest non-emergency waits in 2020 and the longest in 2021, consistent with pandemic-related service disruptions and backlog accumulation.

Conclusion: Emergency MRI prioritization appears effective, with consistently short waiting times for urgent referrals throughout the study period. The primary operational challenge lies within the non-emergency pathway, where substantial variability was observed across gender, nationality, and referring specialty. Given the retrospective observational design, findings are descriptive and hypothesis-generating; observed differences should not be interpreted as confirmed structural inequity without further investigation. More granular non-emergency prioritization, specialty-sensitive capacity planning, and data-driven scheduling strategies may help reduce avoidable delays and promote fairer access to MRI services in public healthcare settings.

## Introduction

Medical imaging plays a pivotal role in modern diagnostics, with magnetic resonance imaging (MRI) being indispensable in both emergency and non-emergency settings. MRI offers superior soft tissue characterization and multiplanar imaging capabilities, making it particularly valuable for the assessment of neurological, musculoskeletal, and selected abdominopelvic conditions. In acute and subacute clinical contexts, MRI is increasingly utilized to support timely diagnosis of conditions such as spinal cord compression, acute ischemic injury, soft tissue infections, and pediatric pathologies, where diagnostic accuracy and tissue differentiation are critical for clinical decision-making [1,2]. In non-emergency settings, MRI is essential for disease staging, treatment planning, and longitudinal monitoring across a wide range of chronic conditions, reinforcing its central role in contemporary clinical workflows [3,4].

Globally, the rapid evolution of imaging technologies has led to improved image quality, reduced acquisition time, and better portability, yet access to timely MRI remains challenging. Delays in MRI exams can result in prolonged hospital stays, higher mortality, and worsened outcomes in acute and chronic conditions [5,6]. In oncology, for instance, delays between diagnostic imaging and biopsy have been associated with tumor progression and poor prognosis [7].

Multiple system-level and patient-level factors contribute to MRI delays, including limited scanner availability, inefficient scheduling systems, weekend service gaps, and hospital overcrowding [5,8]. Further, socioeconomic and demographic variables such as age, gender, insurance status, and nationality have also been identified as potential contributors to unequal access, although these disparities are rarely quantified [9,10].

While imaging delays are recognized as a global healthcare challenge, research examining the specific factors contributing to MRI waiting times within the Saudi Arabian healthcare system remains limited. Prior research has focused on general delays in emergency departments or aggregated imaging workflows [11,12], leaving critical gaps in understanding the drivers of MRI access disparities. Key factors that may influence equitable access to MRI services, including patient gender, nationality, referral source, and clinical urgency, have not been systematically examined in the Saudi context, despite staffing shortages and system inefficiencies being identified as major constraints [13].

This study addresses these knowledge gaps by building upon previous research [14]. This study had one primary objective and three secondary objectives. The primary objective was to quantify differences in MRI waiting times between emergency and non-emergency referral pathways in two Saudi public hospitals over a six-year period (2017-2022). The secondary objectives were (1) to assess waiting time differences by patient gender and nationality; (2) to evaluate variation in waiting times across referring clinical specialties; and (3) to describe annual temporal trends between 2017 and 2022. The study was designed as a descriptive and comparative analysis testing between-group differences in waiting time, not causal pathways or independent predictors. By identifying patterns of variation, the study aims to generate evidence to inform improvements in the efficiency and equity of diagnostic imaging in public healthcare settings.

## Materials and methods

This retrospective cohort study analyzed MRI waiting times using data from two government hospitals in Saudi Arabia, both operating under the Ministry of Health (MOH), with bed capacities of 300 and 450, respectively. These hospitals were selected as data sources because they serve as primary providers of radiological diagnostic services to Saudi nationals within the public healthcare system. The dataset included all patients who underwent MRI examinations between January 1, 2017, and December 31, 2022. Key variables extracted for analysis included patient demographics (gender and nationality), clinical urgency classification (emergency vs. non-emergency), and referring specialty.

Data were retrieved from the Radiology Information System (RIS), a centralized operational database that records MRI scheduling and examination events. Extraction was performed via structured automated queries by trained radiology informatics staff; no manual data entry was performed. All extracted data were anonymized before analysis. Cases with missing key timestamps were excluded from the analysis; a total of 866 cases (approximately 2.4% of initially retrieved records) were excluded on this basis. The unit of analysis was the MRI examination rather than the individual patient; a patient who underwent more than one MRI during the study period could contribute more than one observation.

For each MRI case, key data were collected, including the date of the imaging request, which corresponded to the physician’s decision to request the MRI, and the date the MRI procedure was performed. The dataset was extracted based on system-generated timestamps, a commonly utilized approach in numerous studies for capturing and analyzing key procedural timepoints [15-19]. Additional data points included the case emergency status, classified as either emergency or non-emergency, as well as demographic information such as gender and nationality (Saudi or non-Saudi). Furthermore, each case record included the referring clinical specialty. Cases with incomplete or missing data on key variables, particularly the date of imaging request or the date of the MRI procedure, were excluded from the analysis to ensure the accuracy of waiting time calculations. Emergency status was assigned by the referring clinician at the time of order entry in the RIS, consistent with institutional triage protocols; no post-hoc reclassification of urgency was performed. Referring clinical specialty was defined as the specialty or department recorded for the ordering service in the RIS; cross-referrals were attributed to the specialty that generated the imaging request. Because the analysis relied on routinely collected administrative data, additional clinical variables, including clinical indication, use of contrast, and sedation or anesthesia requirement, were not captured and could not be included in the analysis.

MRI waiting time was calculated as the time interval (in days) between the physician’s imaging request and the date of the MRI procedure, following the definition established by the Western Canada Waiting List Project (WCWL) [20]. This interval reflects the period patients wait to access MRI services after the physician’s request.

Statistical analysis

Descriptive statistics, including means, standard deviations (SD), and median values, were calculated to summarize overall MRI waiting times. Comparative analyses were subsequently performed to assess variations across emergency status (emergency vs. non-emergency), gender, and nationality. Additional subgroup analyses were conducted by referring to the clinical specialty to evaluate specialty-specific waiting times.

Group differences were assessed using Mann-Whitney U tests for group comparisons. Temporal trends were examined descriptively by calculating annual mean and median waiting times from 2017 to 2022 to identify year-to-year variations in service access.

Statistical significance was determined using two-tailed tests, with a p-value < 0.05 considered indicative of significance. All analyses were performed using SPSS version 23.0 (IBM Corp., Armonk, NY) [21].

## Results

Of the 35,934 MRI examinations analyzed, the overall mean waiting time was 24.8 days (SD = 32.61), with a median of 12.93 days. When stratified by emergency status, a statistically significant difference in waiting times was observed (p < 0.001). Emergency examinations (n = 1,128) had substantially shorter waiting times, with a mean of 7.60 days (SD = 18.43) and a median of 1.12 days, compared with non-emergency examinations (n = 34,806), which had a mean waiting time of 25.39 days (SD = 32.83) and a median of 13.73 days.

Gender-based differences in MRI waiting times were further examined across emergency and non-emergency cases. In non-emergency cases, females (n = 18,468) experienced longer waiting times for MRI, with a mean of 26.65 days (SD = 35.15) and a median of 14.94 days, compared with males (n = 16,338), who had a mean waiting time of 22.92 days (SD = 31.26) and a median of 10.76 days; this difference was statistically significant (p < 0.001). For emergency cases, there was no statistically significant difference in waiting times between genders. Female patients (n = 508) had a mean waiting time of 7.97 days (SD = 17.49) and a median of 1.07 days, while male patients (n = 620) had a mean of 6.07 days (SD = 17.49) and a median of 1.04 days. The difference was not significant (p = 0.410), suggesting that gender did not influence waiting time in emergency settings.

Nationality-based differences in MRI waiting times were assessed separately for non-emergency and emergency examinations. Among non-emergency cases, Saudi nationals (n = 31,125) experienced longer waiting times than non-Saudi patients (n = 3,681), with higher mean ranks (16,992.74 vs. 14,021.23; p < 0.001); the corresponding descriptive statistics were a mean of 25.79 days (median = 14.12) for Saudi patients and a mean of 17.51 days (median = 4.16) for non-Saudi patients. In emergency cases, non-Saudi patients (n = 164) had slightly longer waiting times than Saudi patients (n = 964) (mean rank = 641.52 vs. 564.36; p = 0.002), with mean (median) waiting times of 8.32 days (2.39) for non-Saudi patients and 6.69 days (0.99) for Saudi patients.

The analysis identified statistically significant variation in MRI waiting times by emergency status, gender, and nationality (Table 1). Emergency examinations were associated with substantially shorter waiting times than non-emergency examinations. Differences were also observed across gender and nationality within non-emergency cases, though these should be interpreted with caution given the observational design and the absence of adjustment for clinical and case-mix variables.

**Table 1:** MRI waiting times by emergency status, gender, and nationality. ER: emergency room.

Group	N	Mean (SD)	Median	p-value
Emergency status (overall)				<0.001
Non-ER	34,806	25.39 (32.83)	13.73	
ER	1,128	7.60 (18.43)	1.12	
Gender (non-ER)				<0.001
Male	16,338	22.92 (31.26)	10.76	
Female	18,468	26.65 (35.15)	14.94	
Gender (ER)				0.410
Male	620	6.07 (17.49)	1.04	
Female	508	7.97 (17.49)	1.07	
Nationality (non-ER)				<0.001
Saudi	31,125	25.79 (32.65)	14.12	
Non-Saudi	3,681	17.51 (30.48)	4.16	
Nationality (ER)				0.002
Saudi	964	6.69 (17.49)	0.99	
Non-Saudi	164	8.32 (17.49)	2.39	

Temporal trends in MRI waiting times (2017-2022)

Table 2 presents the annual distribution of MRI waiting times for emergency and non-emergency cases from 2017 to 2022. Non-emergency cases consistently experienced longer waiting times across all years. Although some year-to-year variation was observed, there was no clear pattern of sustained increase or decline over the six-year period.

**Table 2:** MRI waiting times by year and emergency status. ER: emergency room.

Year	Non-ER, Mean (SD)	Median	N (Non-ER)	ER, Mean (SD)	Median	N (ER)
2017	26.79 (31.96)	15.10	3,994	6.62 (13.80)	2.03	522
2018	22.07 (28.28)	15.70	5,099	5.09 (10.46)	0.94	155
2019	22.75 (29.75)	15.01	6,134	9.62 (25.23)	0.18	165
2020	17.01 (26.55)	4.95	4,640	9.34 (20.65)	1.97	95
2021	32.79 (37.75)	18.87	7,014	6.14 (22.19)	0.17	105
2022	25.59 (33.82)	11.16	7,925	5.18 (18.87)	0.17	86

Non-emergency waiting times fluctuated over the study period, with the longest mean observed in 2021 (mean = 32.79 days, SD = 37.75) and the shortest in 2020 (mean = 17.01 days, SD = 26.55). These fluctuations may reflect changes in service utilization and scheduling pressures during the COVID-19 period.

Our results also show disparities between the mean and median among non-emergency (non-ER) cases, indicating substantial skewness and variability in waiting time distributions (Table 2). While the mean non-ER wait time declined from 26.79 days in 2017 to 17.01 days in 2020, the corresponding median decreased more sharply, from 15.10 to 4.95 days, suggesting that the majority of cases experienced shorter delays despite a subset encountering prolonged waits. This divergence between central tendency measures was most pronounced in 2020.

MRI waiting times by clinical specialty

The mean waiting times for MRI varied substantially by clinical specialty. Table 3 presents waiting times for selected high-frequency specialties. Pediatric cases experienced the longest average delays (mean = 41.17 days, SD = 45.77), followed by neurology (mean = 35.15 days, SD = 36.05) and urology (mean = 35.05 days, SD = 36.00). Among the specialties shown, orthopedic referrals had the shortest mean waiting time (mean = 28.04 days, SD = 30.48). This variation indicates that MRI waiting times differ across referring specialties, which may reflect differences in case mix, demand volume, scheduling constraints, or prioritization practices, factors that were not directly measured in the present study.

**Table 3:** MRI waiting times by clinical specialty.

Specialty	Mean	Median	SD	N
Surgery	32.65	22.05	35.15	7,363
Urology	35.05	24.98	36.00	9,824
Orthopedic	28.04	19.77	30.48	5,117
Pediatric	41.17	25.13	45.77	1,111
Epilepsy	32.00	25.00	32.45	2,588
Neurology	35.15	25.09	36.05	9,931

## Discussion

This six-year retrospective analysis identified statistically significant variation in MRI waiting times across clinical urgency categories, patient demographics, and referring specialty in two Saudi public hospitals. The prioritization of emergency referrals appears effective in supporting relatively rapid access for urgent examinations. Within the non-emergency pathway, substantial variability was observed across gender, nationality, and referring specialty. These findings highlight operational differences in access patterns that warrant further investigation; the study design does not permit causal inference regarding their mechanisms, nor does it allow formal attribution of the observed differences to structural inequity.

Urgency prioritization and triage structure

The consistently short waiting times for emergency MRI observed across the six-year span (median <2 days) reflect an efficient prioritization system. This aligns with global best practices, where clinical urgency supersedes all other factors in imaging triage [22,23]. These findings are consistent with the preservation of emergency prioritization within the studied hospitals. However, variability in mean wait times across years, including the increase observed in 2019, may reflect intermittent operational constraints related to service demand, workflow inefficiencies, or staffing variation [24,25].

However, the binary classification of urgency (emergency vs. non-emergency) reflects a structural constraint. Non-emergency referrals encompass a wide spectrum of clinical conditions, from routine follow-up to cases with potentially significant diagnostic consequences if delayed. By reducing this heterogeneity to a single category, the system lacks the granularity needed to triage within the non-emergency queue. This approach risks disadvantaging patients with conditions that are time sensitive but not formally urgent, a concern noted in prior critiques of dichotomous scheduling frameworks [20,26]. Multi-level urgency scoring offers an alternative model for prioritizing diagnostic imaging based on nuanced clinical criteria [27,28].

Demographic disparities

This study found statistically significant gender-based differences in non-emergency MRI waiting times, with female patients experiencing longer waits than male patients in this dataset. While this pattern is consistent with broader evidence of sex-based variation in healthcare access, the present study design does not allow determination of whether this reflects structural inequity, differential referral patterns, case-mix variation, or other factors. While this aligns with broader evidence of sex-based inequities in healthcare access [29,30], further investigation is needed. For instance, evidence from the Swedish cataract surgery cohort (2010-2022) reinforces this pattern, revealing that women consistently waited longer than men across all clinical and regional strata, with a mean difference of four days (64 vs. 60 days; p < 0.001) [30]. Even after adjusting for visual acuity, comorbidities, and geography, a linear mixed-effects model confirmed that being male reduced waiting times by 3.3 days (p < 0.001) [30]. Similarly, Canadian data on specialized services highlighted that women constituted 51.7-56.5% of patients awaiting elective surgeries and 51.7% of those awaiting diagnostic tests (e.g., MRI/CT), with 17-29% reporting unacceptable waits [31]. Longer waits correlated strongly with adverse life impacts, including psychological distress and physical deterioration [31].

Contributing factors include systemic scheduling inefficiencies and differential referral patterns. In Sweden, regional disparities in surgeon availability exacerbated delays, disproportionately affecting women in resource-constrained areas [30]. Qualitative studies cited in this analysis further suggest that implicit biases in clinical decision-making, such as differential perceptions of care-seeking assertiveness, may delay prioritization [30,32]. The COVID-19 pandemic amplified these inequities by straining healthcare resources [30], consistent with this study’s observed fluctuations: while 2020 (the initial pandemic peak) saw the shortest non-emergency waits (mean = 17.01 days, SD = 26.55), likely due to temporary service reductions, 2021 recorded the longest waits (mean = 32.79 days, SD = 37.75) as backlogs overwhelmed systems. Addressing these disparities requires targeted interventions, such as standardized referral protocols and equitable resource allocation [30,31].

Differences in non-emergency MRI waiting times by nationality were observed in this dataset, with Saudi nationals accounting for the majority of examinations and recording longer mean and median waiting times than non-Saudi patients. This finding should be interpreted cautiously. In publicly funded systems, waiting times arise when demand exceeds supply, and observed differences may reflect demand concentration, referral composition, and queue dynamics rather than inequitable scheduling practices [33,34]. Without adjustment for clinical indication, case mix, and referral volume, it is not possible to attribute this difference to differential treatment of nationality groups.

Specialty variation and operational solutions

The marked variation in MRI waiting times across clinical specialties, with longer waits for pediatric, neurology, and urology referrals, indicates that the non-emergency MRI queue is not homogeneous and may be shaped by specialty-specific demand, workflow complexity, protocol requirements, and local scheduling practices. This variation may reflect operational constraints rather than intentional prioritization decisions. These disparities align with Canadian healthcare challenges identified by Emery et al. (2009), who found that fewer than half of MRI facilities used explicit, standardized criteria for triage [26]. This inconsistency likely explains why specialties like orthopedics, often prioritized for acute trauma, experienced shorter waits (28.04 days), while pediatric cases faced the longest delays (41.17 days) due to complex needs such as anesthesia coordination and child-specific protocols. Prior literature has suggested that variation in prioritization and wait-list management may contribute to prolonged waiting times in non-urgent MRI pathways [35].

Referral patterns and imaging appropriateness may also influence waiting times. Prior studies have suggested that a proportion of MRI requests may be inappropriate, potentially diverting capacity away from time-sensitive cases. This may be relevant to the longer waits observed for neurology and urology referrals in the present study, although referral appropriateness was not directly assessed in our dataset. Emery et al. reported that 15-30% of MRI requests in Canada may be inappropriate, which may contribute to inefficient use of imaging capacity and longer waits for patients with greater clinical need [26]. In the present study, this issue may be relevant to the extended waiting times observed for neurology and urology referrals (35.15 and 35.05 days, respectively), where high-demand non-urgent examinations may place additional pressure on limited MRI capacity. Without clear referral guidelines or decision-support mechanisms, high-demand specialties may disproportionately burden MRI services, particularly when examinations require specialized protocols or additional coordination.

Addressing these delays requires reforms that balance scanner capacity with referral appropriateness. Emery et al. support the use of validated triage tools, such as the WCWL criteria, to promote more standardized prioritization, while clinical decision-support systems may help improve referral appropriateness and scheduling efficiency [26]. Computerized physician order entry (CPOE) coupled with clinical decision support (CDS) has been identified as a promising approach to improve radiologic service efficiency, reduce inappropriate imaging, and support guideline adherence [36]. Immediate steps could include dedicated MRI slots for pediatric and anesthesia-dependent cases and appropriateness checks aligned with established guidance, such as the Choosing Wisely campaign [37,38]. Future research should link wait times to referral indications and use real-time analytics to align resources more closely with clinical need.

The integration of imaging informatics and artificial intelligence (AI) into radiology is reshaping how diagnostic services are delivered, with particular promise for improving MRI scheduling and addressing access inequities. AI-driven systems, especially when embedded within Radiology Information System-Picture Archiving and Communication System (RIS-PACS) environments, can process imaging data in real time, flagging urgent findings, such as intracranial hemorrhage or acute fractures, and thereby enabling faster, clinically prioritized responses that reduce reporting delays and optimize patient care workflows [39]. Deep learning models have further shown the ability to detect critical abnormalities in MRI scans with high accuracy, making them valuable tools for automated triage and more equitable prioritization of cases [40]. Beyond individual case triage, AI tools offer hospitals advanced analytics capabilities to monitor waitlists, predict demand surges, and identify disparities based on patient demographics or clinical specialty, supporting data-informed decision-making [41-43]. Growing public awareness and acceptance of AI in medical imaging, as reflected in social media discourse, further support its broader adoption within radiology services [44]. These functionalities are particularly relevant in resource-constrained settings, where predictive modeling can help redistribute scanner capacity and expedite imaging for high-risk populations. Evidence from prior health-system studies suggests that standardized triage criteria and digital decision-support tools may improve MRI scheduling efficiency and resource allocation [26]. As Saudi Arabia advances its Vision 2030 digital health strategy, leveraging such technologies presents a timely and evidence-based pathway to reduce systemic delays and ensure fairer access to diagnostic imaging across all population groups.

Based on our findings, we recommend implementing a more granular prioritization system for clinical urgency. We also recommend enhancing system capacity by extending MRI operational hours for routine scans and establishing a centralized digital scheduling platform with real-time analytics. This platform should dynamically redistribute resources based on demand, flag overdue cases, and alert referrers to guideline deviations, while maintaining emergency responsiveness through dedicated staffing and rapid-read protocols.

Limitations

This study has several limitations. First, the analysis was restricted to two public hospitals, limiting generalizability to other regions, private facilities, or hospitals with different staffing and scheduling practices. Second, the study relied on administrative RIS data, and important clinical variables, including clinical indication, case complexity, contrast use, and sedation requirements, were unavailable, precluding multivariable adjustment. Observed group differences may therefore reflect unmeasured confounding. Third, the unit of analysis was the MRI examination, so repeat examinations from the same patient contributed multiple observations. Fourth, only completed examinations were included; canceled, deferred, or never-performed requests were not captured, potentially underestimating the true burden of delayed access. Fifth, the analysis assumed consistent and accurate RIS timestamp recording across both sites throughout the study period; data quality audits were not conducted. Sixth, formal outlier assessment was not performed as a structured analytical step; the presence of extreme waiting time values is instead reflected in the divergence between mean and median values reported throughout the Results, and readers should interpret mean-based statistics with this distributional skewness in mind. Seventh, the study assessed process outcomes only and did not link waiting times to clinical outcomes such as length of stay or disease progression. Finally, hospital-level differences were not separately analyzed. Taken together, these limitations mean the findings should be interpreted as descriptive and hypothesis-generating. Future multicenter studies incorporating richer clinical variables, adjusted analyses, and outcome linkage would substantially strengthen the evidence base.

## Conclusions

This six-year retrospective study found statistically significant variation in MRI waiting times across urgency categories, patient demographics, and referring specialty. Emergency examinations were consistently associated with shorter waiting times, indicating that the emergency prioritization pathway is functioning effectively. Within the non-emergency pathway, differences were observed by gender, nationality, and referring specialty, with the longest delays for pediatric, neurology, and urology referrals.

The findings suggest that the principal constraint lies within the non-emergency queue. While these differences may point toward potential disparities in access, the retrospective observational design and limited clinical variable set do not allow for the determination of underlying causes or formal attribution to structural inequity. The findings should accordingly be interpreted as descriptive and hypothesis-generating. Addressing delays may require more granular prioritization within non-emergency referrals, specialty-sensitive capacity planning, and strengthened referral appropriateness processes. Future research should expand to additional hospitals and regions, incorporate richer clinical and operational variables to enable adjusted analyses, and link waiting times to downstream clinical outcomes and patient experience.
